# Synergic effect of atorvastatin and ambrisentan on sinusoidal and hemodynamic alterations in a rat model of NASH

**DOI:** 10.1242/dmm.048884

**Published:** 2021-05-20

**Authors:** Miren Bravo, Imma Raurell, Aurora Barberá, Diana Hide, Mar Gil, Federico Estrella, María Teresa Salcedo, Salvador Augustin, Joan Genescà, María Martell

**Affiliations:** 1 Liver Unit, Department of Internal Medicine, Hospital Universitari Vall d'Hebron, Vall d'Hebron Institut de Recerca (VHIR), Vall d'Hebron Barcelona Hospital Campus, Universitat Autònoma de Barcelona, Barcelona 08035, Spain; 2 Centro de Investigación Biomédica en Red de Enfermedades Hepáticas y Digestivas (CIBEREHD), Instituto de Salud Carlos III, Madrid 28029, Spain; 3 Department of Pathology, Hospital Universitari Vall d'Hebron, Vall d'Hebron Barcelona Hospital Campus, Barcelona 08035, Spain

**Keywords:** Ambrisentan, Atorvastatin, Endothelin-1, Hepatic stellate cells, Liver sinusoidal endothelial cells, NAFLD-NASH

## Abstract

In non-alcoholic steatohepatitis (NASH), decreased nitric oxide and increased endothelin-1 (ET-1, also known as EDN1) released by sinusoidal endothelial cells (LSEC) induce hepatic stellate cell (HSC) contraction and contribute to portal hypertension (PH). Statins improve LSEC function, and ambrisentan is a selective endothelin-receptor-A antagonist. We aimed to analyse the combined effects of atorvastatin and ambrisentan on liver histopathology and hemodynamics, together with assessing the underlying mechanism in a rat NASH model. Diet-induced NASH rats were treated with atorvastatin (10 mg/kg/day), ambrisentan (30 mg/kg/day or 2 mg/kg/day) or a combination of both for 2 weeks. Hemodynamic parameters were registered and liver histology and serum biochemical determinations analysed. Expression of proteins were studied by immunoblotting. Conditioned media experiments were performed with LSEC. HSCs were characterized by RT-PCR, and a collagen lattice contraction assay was performed. Atorvastatin and ambrisentan act synergistically in combination to completely normalize liver hemodynamics and reverse histological NASH by 75%. Atorvastatin reversed the sinusoidal contractile phenotype, thus improving endothelial function, whereas ambrisentan prevented the contractile response in HSCs by blocking ET-1 response. Additionally, ambrisentan also increased eNOS (also known as Nos3) phosphorylation levels in LSEC, via facilitating the stimulation of endothelin-receptor-B in these cells. Furthermore, the serum alanine aminotransferase of the combined treatment group decreased to normal levels, and this group exhibited a restoration of the HSC quiescent phenotype. The combination of atorvastatin and ambrisentan remarkably improves liver histology and PH in a diet-induced NASH model. By recovering LSEC function, together with inhibiting the activation and contraction of HSC, this combined treatment may be an effective treatment for NASH patients.

## Abstract (precis)

**Summary:** Combining atorvastatin with ambrisentan is safe and effective in reducing intrahepatic resistance and portal hypertension in an experimental model of NASH. This liver histology amelioration highlights a promising therapeutic strategy.

## INTRODUCTION

The prevalence of non-alcoholic fatty liver disease (NAFLD) is increasing worldwide in parallel with other metabolic epidemic disorders, including obesity and type 2 diabetes mellitus (Younossi, 2019). Accordingly, there is an emerging consensus for NAFLD to be defined as MAFLD (Eslam et al., 2020). A subtype of MAFLD, characterized as non-alcoholic steatohepatitis (NASH), is a potentially progressive liver disease that can lead to fibrosis and cirrhosis, with an increased risk of portal hypertension (PH), hepatocellular carcinoma and death (Lindenmeyer and McCullough, 2018). Still, the mechanisms regulating the development and progression of PH in the specific context of NASH are not completely understood.

In cirrhosis, sinusoidal architecture becomes grossly distorted, leading to increased intrahepatic vascular resistance (IHVR) (Bosch et al., 2020). However, increasing clinical and experimental evidence indicates that PH may develop in the early stages of NAFLD when fibrosis is far less advanced or absent (Francque et al., 2010; García-Lezana et al., 2018; Mendes et al., 2012).

Non-parenchymal liver cells contribute to the disruption of sinusoidal homeostasis, increasing IHVR in NASH. Among these cells, liver sinusoidal endothelial cells (LSECs) become dysfunctional and acquire a vasoconstrictor phenotype, releasing decreased levels of vasodilators, such as nitric oxide (NO), and increased levels of vasoconstrictors, such as endothelin-1 (ET-1, also known as EDN-1) (Bravo et al., 2019; Pasarín et al., 2012). Diminished NO production allows hepatic stellate cell (HSC) activation (DeLeve et al., 2008). In this transdifferentiation, HSCs acquire a myofibroblast-like phenotype, with acquisition of alpha-smooth muscle actin increasing their contractility, which may impede sinusoidal flow, augmenting intrahepatic resistance and portal pressure (PP) (Rockey et al., 1992, 1993).

Statins have been shown to improve hepatic endothelial dysfunction (Bosch et al., 2020; Pose et al., 2019). Specifically, chronic treatment with atorvastatin lowered PP by decreasing intrahepatic resistance via the activation of eNOS/NO signaling in different experimental models of cirrhosis (Bravo et al., 2019; Rodríguez et al., 2017; Trebicka et al., 2007).

Another key pathway regulating the HSC contractile phenotype is endothelin signaling. Activated HSCs markedly upregulate endothelin receptors, suggesting an increased sensitivity to ET-1 signal (Yokomori et al., 2001). ET-1 receptor A (ET_A_) stimulation enhances HSC contraction and proliferation, whereas ET-1 receptor B (ET_B_) elicits anti-proliferative activity in HSCs, and induces eNOS activation in LSECs (Liu et al., 2003; Mallat et al., 1996). Furthermore, HSCs experience a significant increase in their sensitivity to ET-1-induced ET_A_ stimulation during transdifferentiation (Reinehr, 2002). This indicates that ET_A_ antagonism would be more advantageous than ET_B_ blocking when trying to reduce PP (Feng et al., 2009).

Ambrisentan, an ET_A_ selective antagonist, has been previously shown to moderately improve PH (Zipprich et al., 2016). However, there is some discrepancy between studies, which probably lies in the different models, doses or routes of administration used (Pitts, 2009).

In the present study, we investigated whether the co-administration of both drugs, atorvastatin and ambrisentan, improves NASH by restoring sinusoidal microcirculation. Specifically, we tested the *in vivo* effect of atorvastatin and ambrisentan on a rat model of NASH with PH and explored their underlying mechanisms on hepatic sinusoidal cells.

## RESULTS

### Treatment effect on body weight gain

**Fig. 1.**
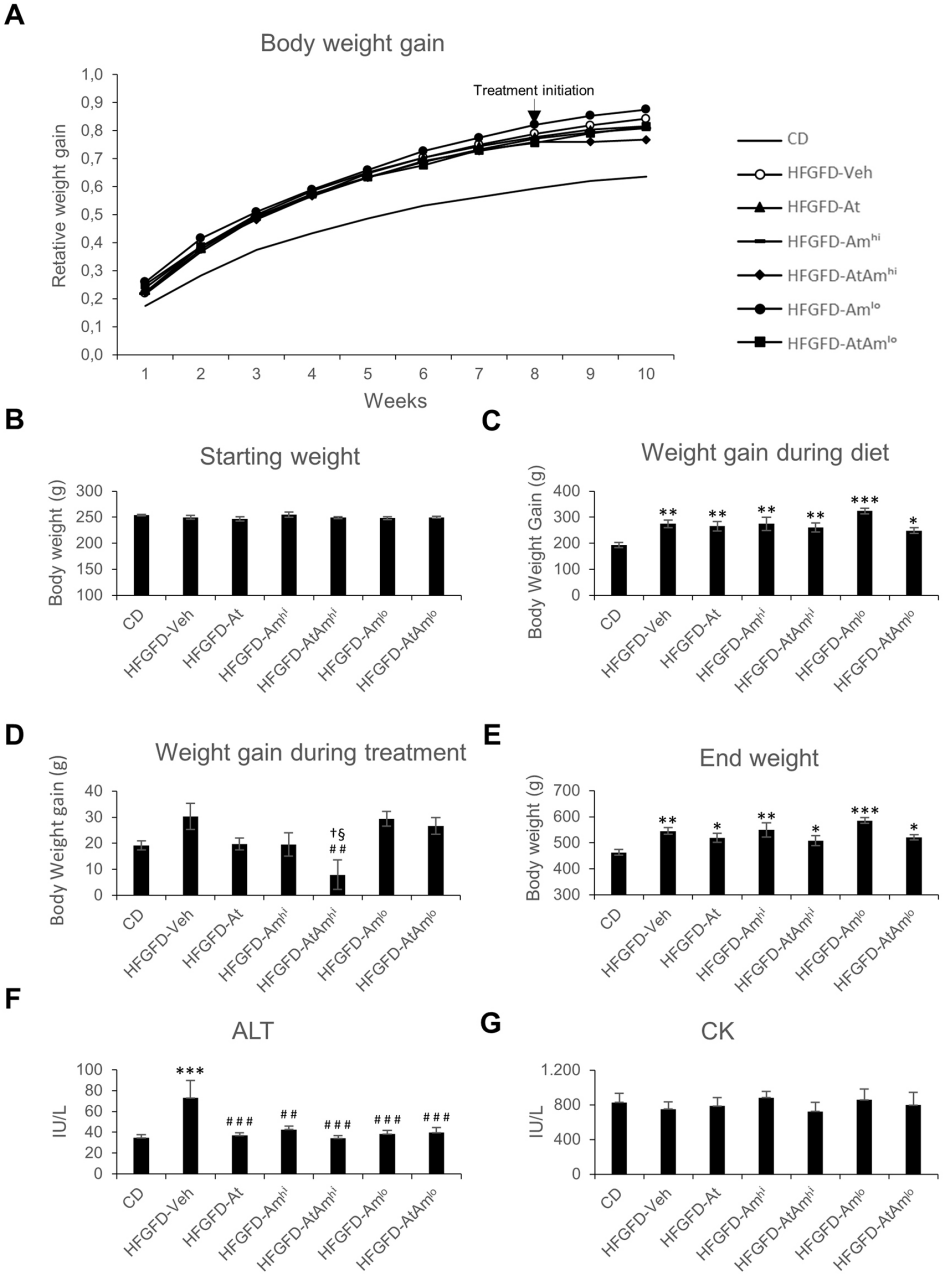
**Body weight gain, ALT and CK values.** (A) Body weight gain at indicated week relative to the starting weight. (B) Starting body weight. (C) Body weight gain during 8 weeks of diet. (D) Body weight gain during 2 weeks of treatment. (E) End body weight. (F) Serum ALT levels. (G) Serum CK levels. Data are mean±s.e.m. CD (*n*=7); HFGFD-Veh (vehicle; *n*=6); HFGFD-At (10 mg/kg/day atorvastatin; *n*=8); HFGFD-Am^hi^ (30 mg/kg/day ambrisentan; *n*=7); HFGFD-AtAm^hi^ (10 mg/kg/day atorvastatin and 30 mg/kg/day ambrisentan; *n*=8); HFGFD-Am^lo^ (2 mg/kg/day ambrisentan; *n*=7); HFGFD-AtAm^lo^ (10 mg/kg/day atorvastatin and 2 mg/kg/day ambrisentan; *n*=8). *P<0.05, **P<0.01, ***P<0.001 versus CD; ^##^*P*<0.01 versus ^###^*P*<0.001 HFGFD-Veh; ^†^*P*<0.05 versus HFGFD-At; ^§^*P*<0.05 versus HFGFD-Am^hi^ (one-way ANOVA).

High fat glucose-fructose diet (HFGFD)-fed groups showed significantly greater body weight gain compared to the control diet (CD)-fed group after 8 weeks of each diet. Weight gain during the next 2 weeks of treatment did not differ between any group of treated and untreated rats, with the exception of the group treated with high-dose ambrisentan combined with atorvastatin (HFGFD-AtAm^hi^), in which body weight gain was significantly lower than that of the vehicle group (HFGFD-Veh, 74% lower increase, *P*=0.001), but also to that of the atorvastatin (HFGFD-At, 60% lower increase, *P*=0.047) and high-dose ambrisentan (HFGFD-Am^hi^, 60% lower increase, *P*=0.036)-treated groups. Furthermore, despite the hypercaloric diet, the HFGFD-AtAm^hi^ group gained 59% less body weight than the CD group during the treatment weeks (Fig. 1). Animals treated with low-dose ambrisentan either alone (HFGFD-Am^lo^) or in combination with atorvastatin (HFGFD-AtAm^lo^) gained weight similar to the other groups during the treatment weeks (Fig. 1).

### Effect of treatments on biochemical parameters

There was a complete absence of both liver and muscular toxicity in all studied groups, considering that no animal exceeded alanine aminotransferase (ALT) and creatine kinase (CK) values shown by the group that received the vehicle treatment. Even with the administration of high-dose ambrisentan there were no signs of liver toxicity, and its combination with atorvastatin did not produce liver or muscle toxicity either (Fig. 1).

A significant increase in serum ALT levels was observed in HFGFD-Veh rats compared to CD animals. This elevation was abolished with all treatment groups, as well as with a reduction in the dose of ambrisentan (Fig. 1).

Table S1 shows serum biochemical parameters analysed in all samples. The combination of ambrisentan and atorvastatin lowered serum cholesterol levels compared to the CD group, but not in comparison with the HFGFD-Veh group.

### Combined treatment markedly improves liver hemodynamics in rats with NASH

Systemic circulation was not affected by the diet and neither by different treatments at any doses.

As expected, the PP was higher in HFGFD-Veh rats than in CD rats (27.8% increase, *P*<0.001). This was accompanied by a significant increase in IHVR and decreased portal blood flow (PBF) (Table 1). In these rats, atorvastatin monotherapy caused a significant decrease in PP (HFGFD-At, 12.8%, *P*=0.001), associated with a moderate reduction in IHVR, whereas PBF remained unchanged.

**Table 1.**
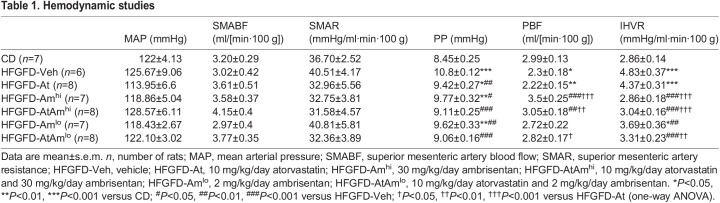
Hemodynamic studies

Treatments with high-dose ambrisentan, alone or in combination, achieved a significant decrease in PP (HFGFD-Am^hi^, 9.5%, *P*=0.011; HFGFD-AtAm^hi^, 15.7%, *P*<0.001) compared to the HFGFD-Veh group. This reduction in PP was due to a marked decrease in IHVR produced by ambrisentan. However, high-dose ambrisentan groups also showed a significant increase in PBF compared to the HFGFD-Veh group.

The reduction in IHVR of the HFGFD-Am^hi^ group was significantly greater than that of the HFGFD-At group. However, this did not translate into a greater effect on PP, probably due to the increase in PBF produced by ambrisentan at high dose.

Ambrisentan dose reduction, both in monotherapy and in combination, maintained the beneficial effects, significantly decreasing PP (HFGFD Am^lo^, 10.9%, *P*=0.004; HFGFD-AtAm^lo^, 16.1%, *P*<0.001), together with IHVR compared to the vehicle. But, in addition, it prevented portal flow increase, so that both HFGFD-Am^lo^ and HFGFD-AtAm^lo^ groups did not show differences in PBF values compared to the HFGFD-Veh group. Therefore, we used the lower dose for the histological and molecular analysis.

The combination of atorvastatin and low-dose ambrisentan achieved a greater general improvement in liver hemodynamics than that obtained with each of the drugs alone. As a consequence, the HFGFD-AtAm^lo^ group showed PP, PBF and IHVR values comparable to those of the CD group (Table 1).

### Combination treatment reverses histological NASH

**Fig. 2.**
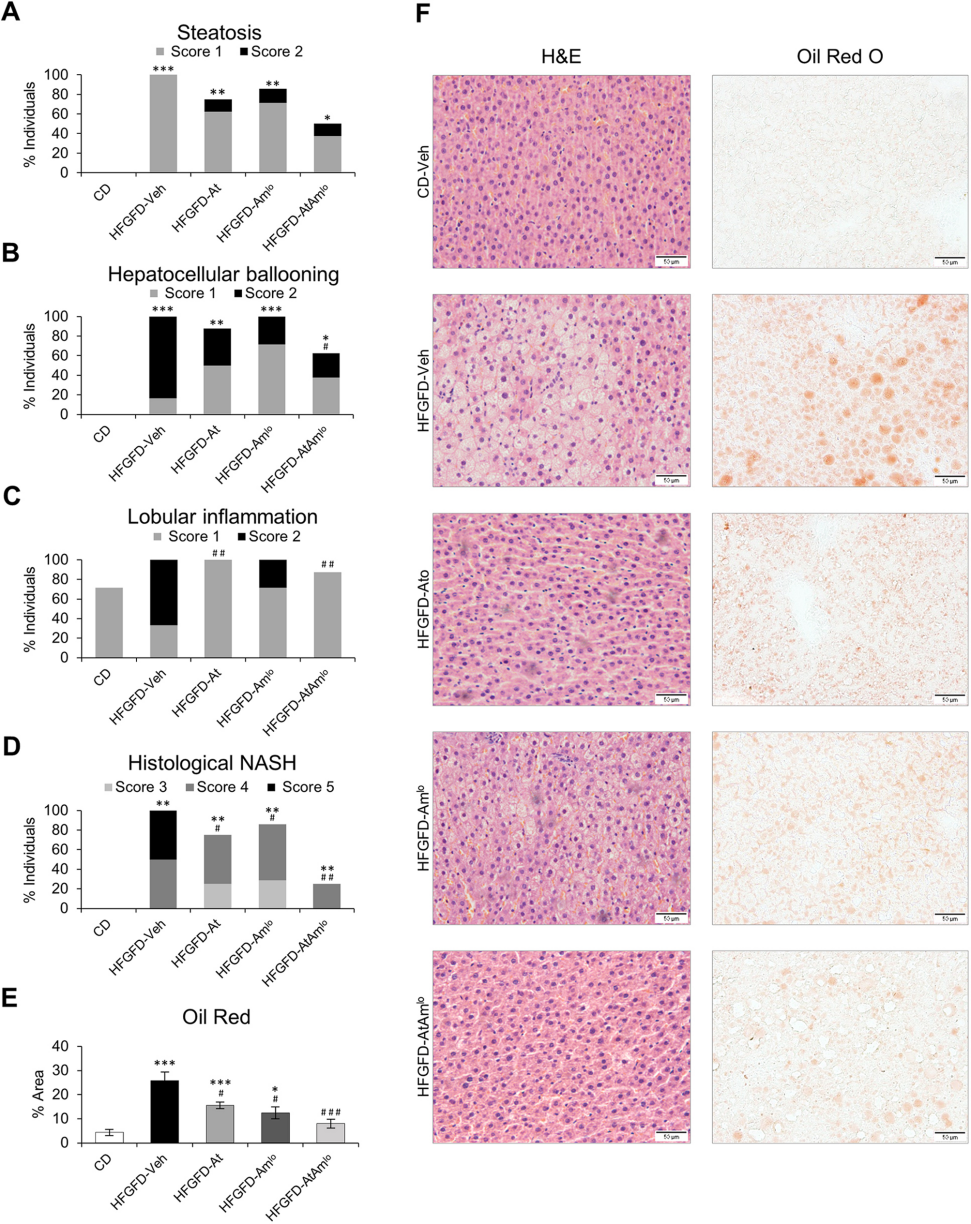
**Histological evaluation of NASH activity following the NASH-Clinical Research Network system.** (A-C) Bar graphs represent the percentage of individuals presenting steatosis (A), ballooning (B) and inflammation (C) in CD (*n*=7; HFGFD-Veh, *n*=6; HFGFD-At, *n*=8; HFGFD-Am^lo^, *n*=7; and HFGFD-AtAm^lo^, *n*=8). Each colour represents the percentage of individuals with the corresponding score (score 1, gray; score 2, black). (D) Bar graphs show the percentage of individuals with histological NASH represented with different NAS scores (score 3, light gray; score 4, dark gray; or score 5, black). (E) Bar graph representing the quantification of liver lipid by Oil Red O staining in liver sections. (F) Representative images (20× magnification) of liver sections stained with hematoxylin-eosin and Oil Red O used to perform the histological evaluation. *P<0.05; **P<0.01; ***P<0.001 versus CD; ^#^*P*<0.05; ^##^*P*<0.01; ^###^*P*<0.001 versus HFGFD-Veh (Mann–Whitney *U-*test).

HFGFD caused histological NASH, defined as the concurrence of steatosis, hepatocellular ballooning and lobular inflammation (Fig. 2; Fig. S1). HFGFD-fed animals did not develop fibrosis (Fig. S2).

Hepatic steatosis, analysed by the CRN system, persisted in most groups after the different treatments, although the combination of atorvastatin and ambrisentan markedly reduced the percentage of individuals with this feature (Fig. 2A,F). Moreover, Oil Red O staining showed a significant decrease in the percentage of the steatosis area with all treatments. In the case of the combined treatment, the percentage of steatosis was similar to that of the control group (Fig. 2E,F). Hepatocellular ballooning improved significantly in the HFGFD-AtAm^lo^ group (Fig. 2B,F), and the lobular inflammation score improved with atorvastatin treatment both in monotherapy and in combination with ambrisentan (Fig. 2C,F).

All three parameters correlated with serum transaminase levels in individual samples. Although the correlation between the steatosis score and ALT levels was not significant, Oil Red O staining did significantly correlate in these same individuals (Fig. S3).

Accordingly, all animal groups receiving treatment showed lower NAFLD activity score (NAS) values than those achieved by the vehicle group. NAS also significantly correlated with serum ALT levels (Fig. S3E). Furthermore, both atorvastatin and ambrisentan reduced the percentage of rats with histological NASH, although only the combination of both achieved a superior histopathological improvement, reversing histological NASH in 75% of individuals (Fig. 2D).

### Atorvastatin improves insulin sensitivity

Fasting insulin levels increased significantly in the HFGFD-fed group, as well as insulin resistance [homeostatic model assessment of insulin resistance (HOMA-IR)]. Atorvastatin treatment alone or in combination (HFGFD-At and HFGFD-AtAm^lo^) significantly reduced serum insulin levels, and both groups showed a remarkable insulin sensitivity recovery, although only the group with the combination achieved a statistically significant decrease in the HOMA-IR index. Otherwise, in the HFGFD-Am^lo^ group, both insulin levels and the HOMA-IR index were similar to those of the vehicle group (Table 2).

**Table 2.**
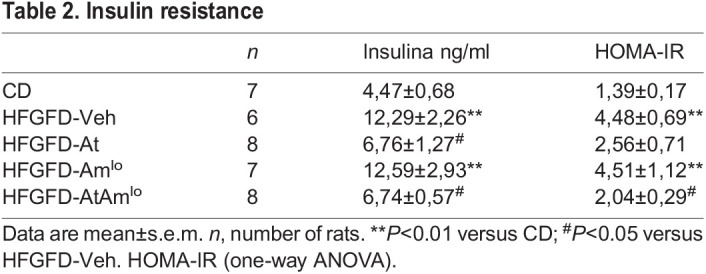
Insulin resistance

### Recovery of the intrahepatic vasoactive mediators by treatment combination

**Fig. 3.**
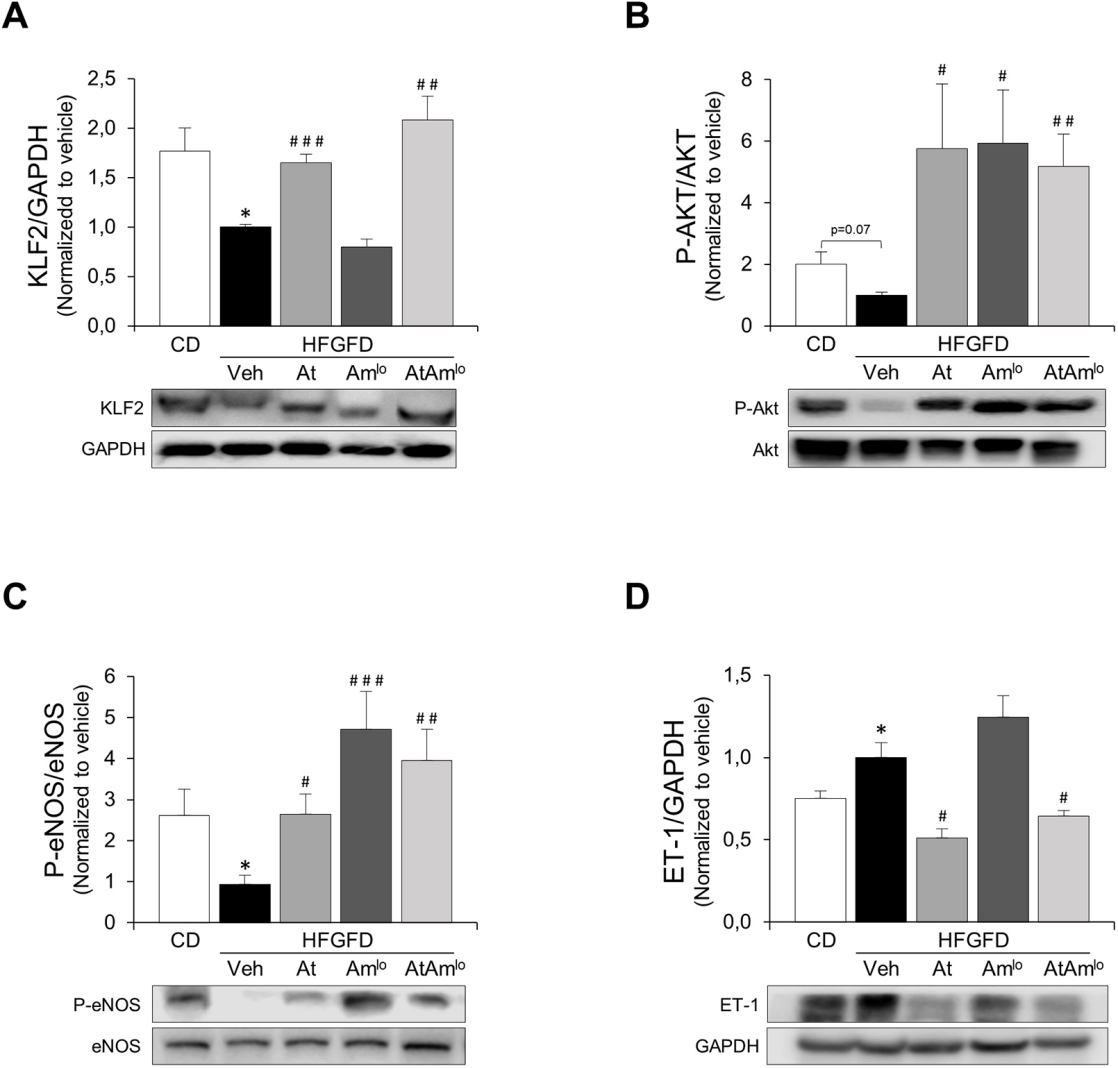
**Intrahepatic expression of vasoactive modulators.** (A-D) Bar graphs show the quantification of KLF2 (A), P-Akt/Akt (B), P-eNOS/eNOS (C) and ET-1 (D) using GAPDH as a loading control in CD, *n*=7; HFGFD-Veh, *n*=6; HFGFD-At, *n*=8; HFGFD-Am^lo^, *n*=7; and HFGFD AtAm^lo^, *n*=8. Protein levels are normalized with the HFGFD-Veh group and expressed as mean±s.e.m. Repesentative western blot bands are shown below. *P<0.05 versus CD; ^#^*P*<0.05, ^##^*P*<0.01, ^###^*P*<0.001 versus HFGFD-Veh (unpaired two-tailed Student's *t*-test).

Western blot analysis revealed a decreased intrahepatic protein expression of Kruppel-like factor 2 (KLF2) (Fig. 3A), together with Akt and eNOS (also known as Nos3) phosphorylation reduction in HFGFD-Veh rats compared to the CD group (Fig. 3B,C). These results suggest microvascular dysfunction as the underlying mechanism of increased IHVR in the model. Atorvastatin treatment increased KLF2, phosphorylated endothelial nitric oxide synthase (P-eNOS) and phosphorylated protein kinase B (P-Akt) liver content (Fig. 3A-C). Ambrisentan administration significantly increased eNOS and Akt phosphorylation (Fig. 3A, Fig. 2B), but did not affect the expression of KLF2 (Fig. 3C). As expected, the combination of both treatments increased KLF2, P-eNOS and P-Akt intrahepatic levels (Fig. 3A-C).

Moreover, intrahepatic ET-1 expression was significantly elevated in HFGFD-Veh rats compared to the CD group. In HFGFD-At and HFGFD-AtAm^lo^ groups, the protein expression levels of this vasoconstrictor decreased significantly, but not in HFGFD-Am^lo^ group (Fig. 3D).

Fig. S4 shows the complete western blot images used for the quantification of the protein immunoblots.

### Ambrisentan increases eNOS activation in LSEC

**Fig. 4.**
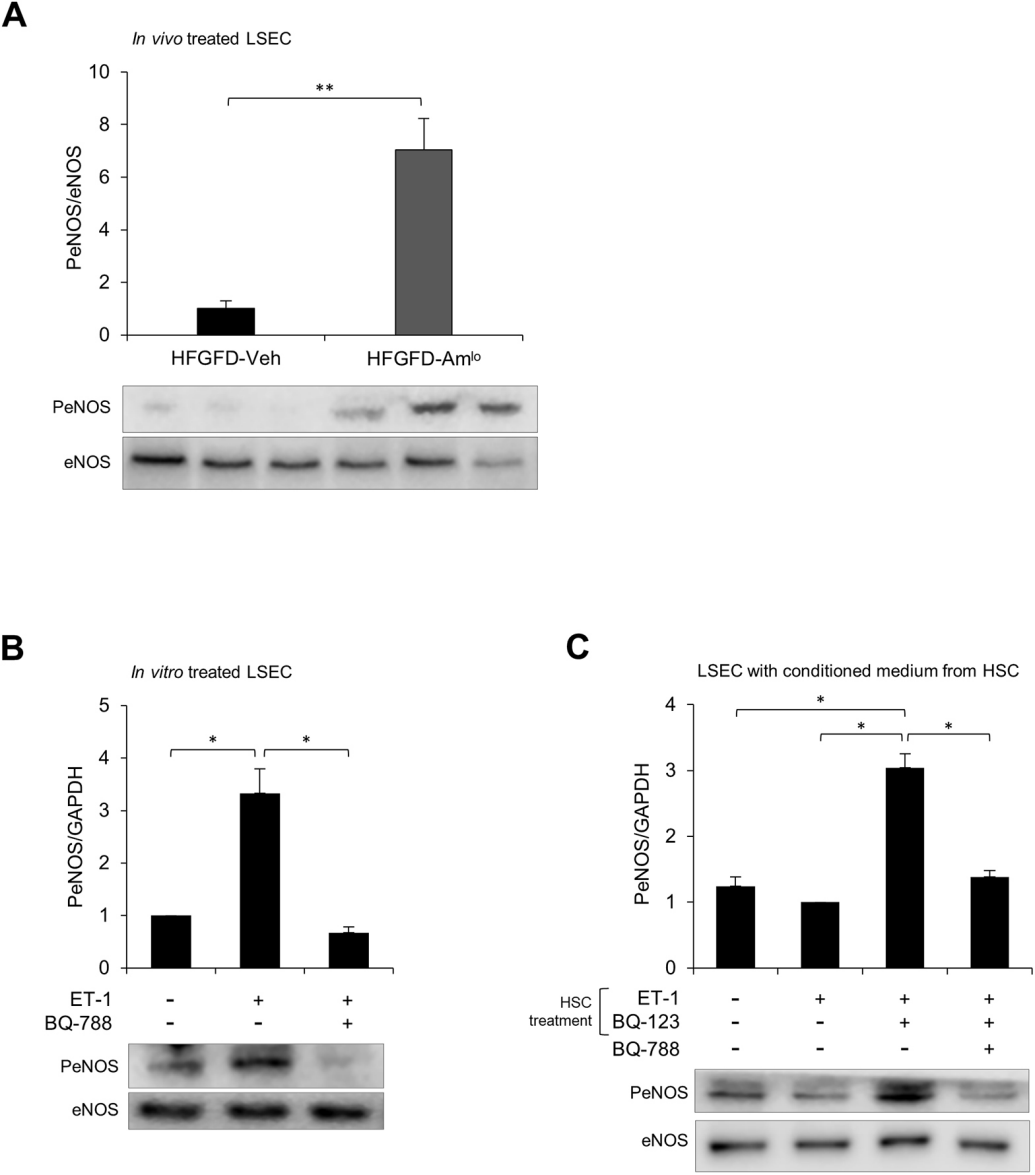
**Effect of ET-1 in eNOS activation in LSECs.** (A-C) Bar graphs show quantification of P-eNOS/eNOS ratio from LSECs (A) isolated from HFGFD-Veh (*n*=3) and HFGFD-Amlo (*n*=3) normalized to the HFGFD-Veh group. (B) LSECs treated with BQ-788 (1 µM) or vehicle for 15 min and exposed to ET-1 (10 nM) for 30 min. (C) LSECs treated with BQ-788 (1 µM) or vehicle for 15 min, and exposed for 30 min to conditioned medium from HSCs treated with BQ-123 (10 µM) or vehicle for 10 min, and incubated with ET-1 (10 nM) for 10 min. The immunoblot shown is representative of three different experiments, each performed with cells from a different isolation. *P<0.05; **P<0.01 (unpaired two-tailed Student's *t*-test).

As ambrisentan has not yet been reported to increase eNOS phosphorylation levels, we decided to corroborate this result directly in LSECs isolated from ambrisentan-treated rats. These cells showed a significant increase in the expression of P-eNOS compared to LSEC isolated from the vehicle group (Fig. 4A).

To further understand the ambrisentan mechanism for inducing eNOS activation, we decided to analyse the ET-1 pathway in LSECs. Exposure of freshly isolated LSECs to ET-1 led to a significant increase in eNOS phosphorylation (Fig. 4B). Besides, blockade of the ET_B_ receptor with the specific receptor antagonist BQ-788 inhibited ET-1-mediated eNOS phosphorylation, verifying that in LSECs, ET-1-mediated eNOS activation derives from ET_B_ signaling (Fig. 4B).

ET-1-induced eNOS activation was abolished when LSECs were exposed to conditioned medium from HSCs previously treated with ET-1, showing no increases in P-eNOS levels in LSECs (Fig. 4C). However, when HSCs were previously treated with the ET_A_ specific receptor antagonist BQ-123, ET-1 remained capable of inducing eNOS phosphorylation in LSECs exposed to HSC-conditioned medium. As expected, LSEC pre-treatment with BQ-788 reverted eNOS activation in this condition (Fig. 4C).

### Atorvastatin reduces HSC contractility and ambrisentan blocks ET-1 induced contraction

**Fig. 5.**
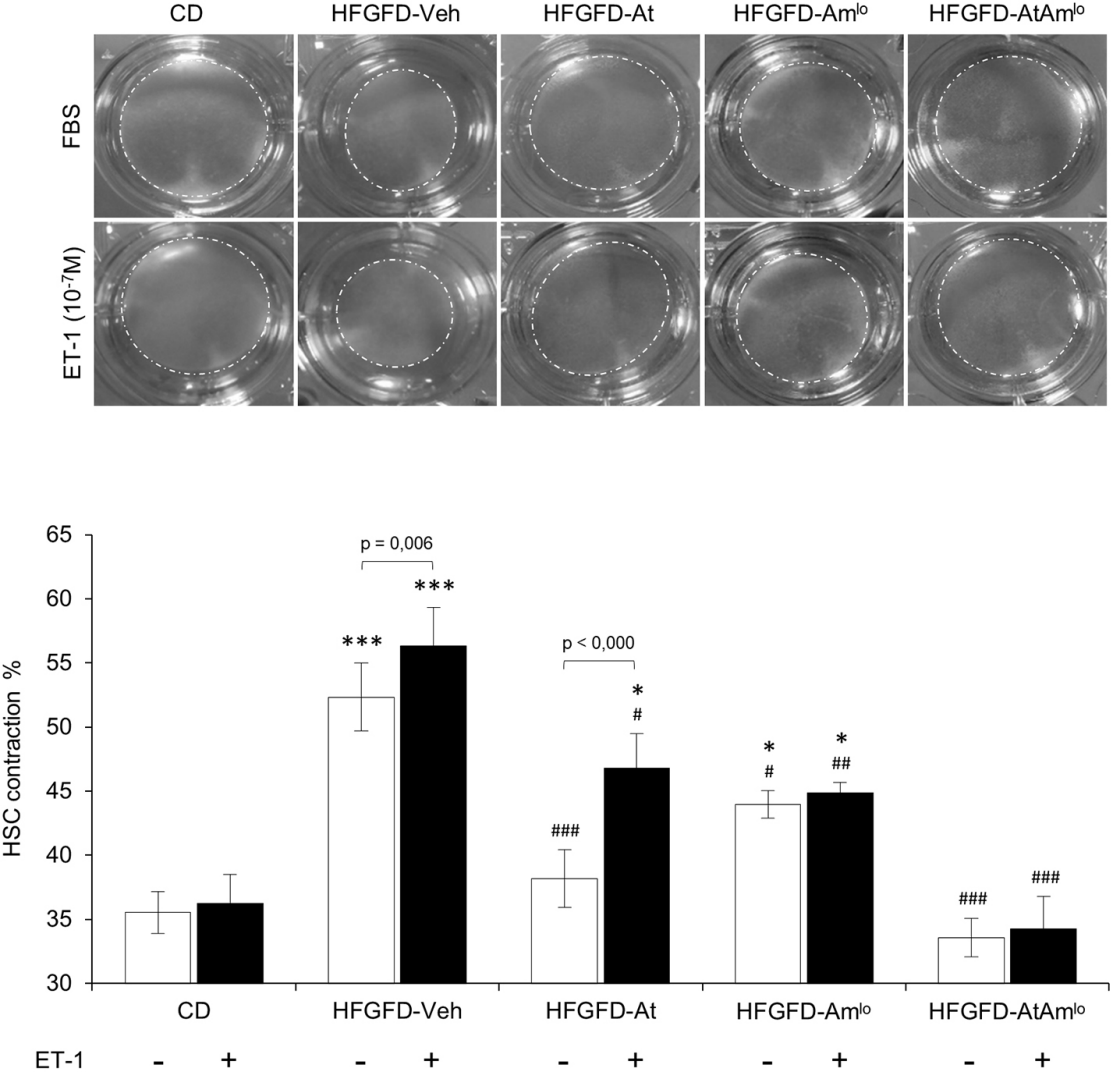
**HSC contraction on collagen gel lattices.** Contraction is expressed as the percentage of initial gel area as mean±s.e.m. All data are from experiments using three collagen lattices for each condition with HSCs isolated from CD, *n*=3; HFGFD-Veh, *n*=4; HFGFD-At, *n*=5; HFGFD-Am^lo^, *n*=4; and HFGFD AtAm^lo^, *n*=4. *P<0.05; ***P<0.001 versus CD; ^#^*P*<0.05; ^##^*P*<0.01; ^###^*P*<0.001 versus HFGFD-Veh (one-way ANOVA).

To investigate whether IHVR reduction obtained by oral treatments is due to HSC relaxation, we performed contraction assays in the basal situation and in response to ET-1, with HSCs isolated from the different groups (Fig. 5).

In basal conditions, HSCs isolated from the HFGFD-Veh group showed a significantly higher percentage of contraction than cells from the CD group. The contractile capacity significantly decreased in cells isolated from all treatment groups, but especially in those from HFGFD-At and HFGFD-AtAm^lo^ animals, the contraction percentage of which was comparable to that shown by CD group cells.

*In vitro* treatment with exogenous ET-1 generated a superior contraction of the collagen gel in HSCs of HFGFD-Veh rats, whereas cells of CD rats maintained their low percentage of contraction. Results showed increased contraction of HSCs from HFGFD-At in response to ET-1, whereas cells from HFGFD-Am^lo^ exhibited a completely blocked ET-1-mediated contractile response. HSCs from the HFGFD-AtAm^lo^ group showed an almost identical percentage of collagen gel contraction to those of the control group, both in standard conditions and after ET-1 treatment (Fig. 5).

### Combination treatment reverses HSC pro-contractile and pro-fibrogenic profile

**Fig. 6.**
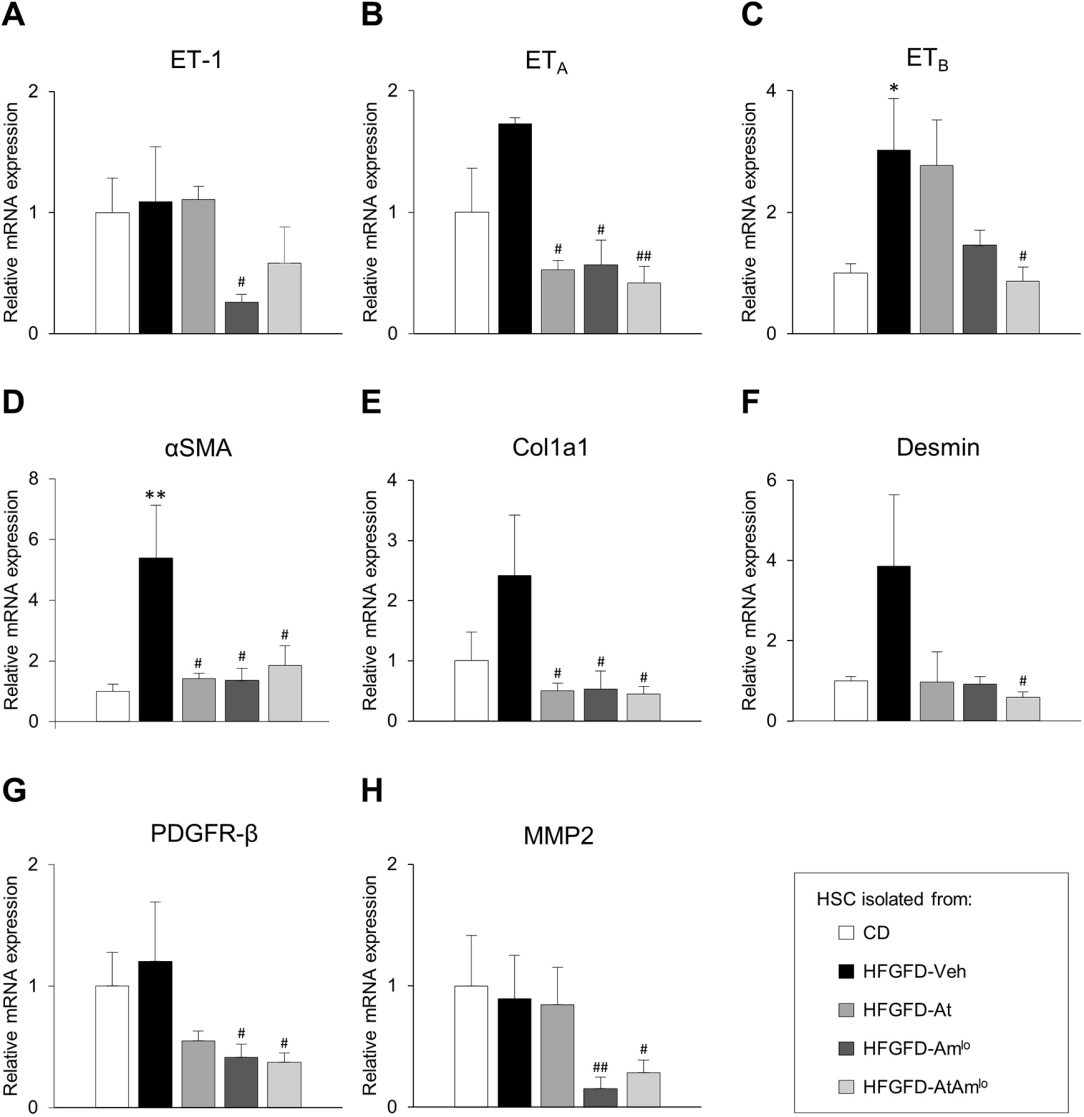
**Expression of pro-contractile and pro-fibrotic markers in HSCs.** Relative quantification of mRNA expression of endothelin-1 (ET-1), endothelin receptor A (ET_A_), endothelin receptor B (ET_B_), alpha-smooth muscle actin (αSMA), collagen type I alpha 1 (Col1a1), desmin, platelet-derived growth factor receptor-beta (PDGFR-β) and matrix metalloproteinase-2 (MMP2) by qRT-PCR in HSCs isolated from CD (*n*=4), HFGFD-Veh (*n*=4), HFGFD-At (*n*=5), HFGFD-Amb^lo^ (*n*=4) and HFGFD-AtAm^lo^ (*n*=5). GAPDH was used as an endogenous control and the results were normalized to HSCs from CD. Data are mean±s.e.m. *P<0.05; **P<0.01 versus CD; ^#^*P*<0.05; ^##^*P*<0.01 versus HFGFD-Veh (one-way ANOVA).

ET-1 mRNA levels remained unchanged in HSCs from HFGFD-Veh rats compared to the control group. Ambrisentan treatment significantly decreased ET-1 gene expression in these cells (Fig. 6A). Concerning ET-1 receptors, HFGFD-Veh-isolated HSCs showed increased levels of both receptor types, ET_A_ and ET_B_, compared to HSCs from the CD group (Fig. 6B,C). All treatment groups showed a significant reduction in ET_A_ mRNA levels (Fig. 6B). However, ET_B_ expression was only significantly downregulated in HSCs from the HFGFD-AtAm^lo^ group compared to those of the HFGFD-Veh group (Fig. 6C).

HSCs isolated from HFGFD-Veh exhibited increased expression of αSMA (also known as Acta2), Col1a1 and desmin mRNA, suggesting an activated phenotype in these cells (Fig. 6D-F). HSCs isolated from both atorvastatin- and ambrisentan-treated animals showed a significant reduction in αSMA and Col1a1 levels, and a marked desmin downregulation. The combined treatment achieved a superior amelioration of the HSC phenotype, reducing significantly the levels of these three activation-associated markers (Fig. 6D-F).

The expression levels of platelet-derived growth factor receptor β (PDGFR-β) and matrix metalloproteinase 2 (MMP2) in HSCs were not modified in the HFGFD-Veh group compared to the CD group. However, cells from the HFGFD-Am^lo^ and HFGFD-AtAm^lo^ groups exhibited significantly lower levels of both genes compared with HSCs from the HFGFD-Veh group (Fig. 6G,H).

## DISCUSSION

In this study, we showed that the combination of atorvastatin and ambrisentan normalizes liver hemodynamics, reducing IHVR and PP in rats with histologically diagnosed NASH.

PH and its derived complications represent the main cause of liver failure and transplantation in patients with advanced chronic liver diseases (García-Pagán et al., 2012). However, PH is not solely the consequence of cirrhosis, as sinusoidal microvascular dysfunction contributes also to increase IHVR, and consequently, sinusoidal portal pressure, which may impact disease progression in MAFLD (Ryou et al., 2020). Consistent with these data, our NASH model presents PH, together with a marked microvascular dysfunction characterized by decreased eNOS activation in LSECs and increased HSC contraction.

Decreased eNOS activity is probably the result of reduced expression of KLF2, a transcription factor that confers endothelial protection in response to shear stress, inducing NO production (Marrone et al., 2015). Our results are in agreement with previous studies that evidence a lowered PP mediated by a significant upregulation of KLF2 protein expression and eNOS phosphorylation in livers from atorvastatin-treated animals (Bravo et al., 2019; Rodríguez et al., 2017; Trebicka et al., 2007). On the other hand, insulin also plays a very important role in the regulation of intrahepatic eNOS activity through the IRS/PI3K/Akt pathway (García-Lezana et al., 2018; Pasarín et al., 2011). Our model evidenced insulin resistance improvement by atorvastatin treatment, suggesting that atorvastatin-induced P-Akt increase is, at least partially, mediated through the insulin vasodilator response recovery. However, we cannot rule out other mechanisms, as statins have been shown to promote insulin-independent Akt-mediated eNOS phosphorylation (Trebicka and Schierwagen, 2015; Trebicka et al., 2007).

Atorvastatin-induced endothelial function restoration was accompanied by a lower production of ET-1 at the intrahepatic level in our model. Our data support the hypothesis that atorvastatin decreases HSC contraction and consequently reduces IHVR (Trebicka et al., 2007). In accordance, our NASH model showed enhanced HSC contraction, correlated with increased αSMA (pro-contractile marker) expression, and this was also reversed by oral administration of atorvastatin.

On the other hand, ambrisentan also showed to be effective in reducing PP. Endothelin receptor antagonists (ERAs) have been indicated for the treatment of pulmonary arterial hypertension. Currently, several studies have demonstrated the ability of these drugs to modulate liver hemodynamics in cirrhotic models, although with discordant results due to the great variation in ERA type and administered dose (Cavasin et al., 2010; Feng et al., 2009; Kojima et al., 2000). Our results demonstrate clearly that ambrisentan at 2 mg/kg/day shows significant intrahepatic beneficial effects. Increasing the dose does not improve the effect, but leads to the appearance of unwanted side effects, such as an increase of PBF.

Long-term administration of some ERAs, such as bosentan, have been repeatedly associated with acute and severe liver injury (Humbert et al., 2007). Yet, ambrisentan has not been associated with hepatotoxicity (Galiè et al., 2008; Kenna et al., 2015), not even in patients who had previously discontinued treatment with bosentan due to alterations in aminotransferase levels (McGoon et al., 2009). In accordance with these data, none of the animals in our study showed liver toxicity, not even those receiving the high dose of ambrisentan, and all treatments normalized ALT levels. These results reinforce the idea that ambrisentan is safe and not hepatotoxic. Nonetheless, more studies are needed to validate its safety in more advanced liver disease.

Animals treated with atorvastatin and ambrisentan combined showed decreased serum cholesterol levels compared to the CD group. This suggests that the combined treatment might be beneficial in controlling dyslipidaemia in NASH patients. However, we cannot fully affirm this effect as our model does not show hypercholesterolemia.

In the case of ambrisentan treatment, PP reduction was accompanied by the improvement of the HSC phenotype by blocking the ET_A_ receptor, which is exclusively found in these cells. Ambrisentan also reduced the contraction of HSC but, in particular, it completely inhibited their exacerbated contractile response to exogenous ET-1. Thus, ambrisentan might directly decrease intrahepatic vasoconstriction response and thus decrease intrahepatic vascular tone.

As expected, there was no improvement in insulin sensitivity or increased KLF2 expression in ambrisentan-treated animals. However, we observed increased intrahepatic eNOS phosphorylation levels. We even corroborated this increased P-eNOS level in LSECs freshly isolated from *in vivo* ambrisentan-treated rats. In the present study, we demonstrate for the first time that ambrisentan has an indirect effect on LSECs, increasing eNOS activation. ET_A_ receptor blockade in HSCs by ambrisentan increases the bioavailability of ET-1, which binds to ET_B_ receptors located in LSECs. As described by others, and as we demonstrate in this study, ET-1 stimulation of ET_B_ receptors in LSECs increases eNOS phosphorylation and, thus, NO synthesis, leading to vasodilatation (Liu et al., 2003). Therefore, ambrisentan might indirectly contribute to sinusoidal microvascular function improvement.

Consistent with the results obtained from the atorvastatin or ambrisentan monotherapies, combination treatment showed synergistic benefits. Thus, a clear restoration of sinusoidal microvascular function, together with significant IHVR and PP reduction, was exhibited in the double-treated rats.

HSC contractile response to ET-1 has been shown to increase with the progression of liver injury, being proportional to its activation degree (Kawada et al., 1993; Rockey and Weisiger, 1996). In accordance, our NASH model showed increased levels of intrahepatic ET-1, and an aggravated HSC contraction. Moreover, HSCs from our model had increased gene expression levels of both ET-1 receptors, which would explain the exacerbated contraction when incubated with this vasoconstrictor. HSCs isolated from atorvastatin-treated rats also exhibited this increased contraction in response to exogenous ET-1. However, the HSCs of rats treated with ambrisentan did not increase their contraction when incubated with ET-1, demonstrating that much of the contraction exerted by the HSCs in response to ET-1 is via ET_A_. The combination of both drugs maintained HSC contractility indistinguishable from that from healthy animals, and like these, they did not show a contractile response when incubated with exogenous ET-1. These results suggest an important role for HSCs and their response to ET-1 in the pathophysiology of PH in NASH.

The combination of atorvastatin and ambrisentan was the only treatment capable of significantly reducing the expression levels of both ET_A_ and ET_B_ receptors. This shows once again a close relationship between HSC activation and the ET-1 system. Both atorvastatin and ambrisentan obtained similar effects with respect to improving HSC phenotype, as indicated by expression reduction of activation markers (αSMA, Col1a1 and PDGFR-β). However, only the combination of both significantly reduced desmin expression, demonstrating a synergistic effect of both drugs on HSCs.

ET-1 has a prominent contractile effect on HSCs, which can contribute to PH, but also promotes their proliferation and migration in early stages of the disease (Friedman, 2008). Our results show that treatment with ambrisentan inhibits the expression of MMP-2 induced by ET-1. This effect was described previously by *in vitro* treatment of activated HSCs with BQ-123 (Koda et al., 2006). Here, we confirm that this also occurs with *in vivo* treatment, suggesting that decreasing MMP2 expression by ET_A_ antagonisms could prevent the degradation of the normal subendothelial matrix and its subsequent replacement by a non-functional extracellular interstitial matrix. These findings show the need for additional research in more advanced models of NASH to analyse the effect of the combination of these drugs on fibrogenesis. We clearly acknowledge that our NASH model lacks fibrosis generation and this is a limitation of our study.

Finally, the combination treatment markedly improved liver histopathology. Both drugs decreased steatosis area, but especially, individuals from the HFGFD-AtAm^lo^ group showed an Oil Red O-stained area similar to that of the CD group. Atorvastatin, alone or in combination, significantly improved lobular inflammation. However, ballooning improved significantly only with the combined treatment. So, despite the fact that all treatments reduced the NAS score, the combination achieved a superior improvement, reversing histological NASH in 75% of individuals. Although we hypothesize that atorvastatin and ambrisentan act directly in LSECs and HSCs, and that eventually these effects might indirectly collaborate in improving liver histology, a direct effect on hepatocyte function cannot be ruled out. Furthermore, there may be off-target effects involving other cell types, which brings out the need for additional studies to determine these complex interactions.

In conclusion, this study suggests that the combination of atorvastatin and ambrisentan normalizes intrahepatic vascular tone, recovering LSEC function, together with inhibiting the proliferation and contraction of HSCs. This turns into amelioration of liver histology and PH in the early stages of NASH, and consequently, it might prevent disease progression. These findings support the idea that this combination could be a safe and effective treatment for patients with NASH. The potential long-term use of the combination treatment explored here in patients with NAFLD/NASH is very attractive for slowing the progression of the disease, considering the low cost of the drugs, a very low toxicity profile and possible effects in human NAFLD physiopathology. Before that, first, proof of a beneficial effect in fibrotic NASH models would be required and, from there, a demonstrated clinical effect in a clinical trial would also be needed.

## MATERIALS AND METHODS

### Animal model

All procedures were conducted in accordance with European Union Guidelines for Ethical Care of Experimental Animals (EC Directive 86/609/EEC for animal experiments) and approved (file number: 10,989) by the Animal Care Committee of the Vall d'Hebron Institut de Recerca (VHIR, Barcelona, Spain) and conducted in the animal facilities of VHIR.

Male Sprague-Dawley OFA rats (Charles River Laboratories, L'Arbresle, France) weighing 200-220 g were used for our previously described diet-induced NASH model (García-Lezana et al., 2018). Rats were housed under 12h light/dark cycle at constant temperature and humidity. They were fed *ad libitum* for 8 weeks with a HFGFD or CD. The HFGFD consisted of 30% fat (butter, coconut oil, palm oil and beef tallow), with mainly saturated fatty acids (5.73 Kcal/g), supplemented with cholesterol (1g/Kg) (Ssniff Spezialdiaten GmbH, Soest, Germany), and a beverage of glucose-fructose (42g/L, 45% glucose-55% fructose). The CD consisted of a grain-based chow that consisted of 4.8% fat (3.43 Kcal/g) (Safe-150, SAFE, Augy, France) and tap water. Body weight and food consumption were monitored weekly.

### Drug administration/treatments

Eight-week HFGFD-fed rats received daily oral doses of the corresponding drug or vehicle for 2 weeks. Atorvastatin (10 mg/kg/day, Almirall, Barcelona, Spain) (HFGFD-At), 30 mg/kg/day (HFGFD-Am^hi^) or 2 mg/kg/day (HFGFD-Am^lo^) ambrisentan (GlaxoSmithKline, Dublin, Ireland), a combination of the same dose of atorvastatin with both doses of ambrisentan (HFGFD-AtAm^hi^ and HFGFD-AtAm^lo^) or equivalent volume of water (HFGFD-Veh) were administered by gastric gavage. Each group continued to have access to the original diet during the entire treatment period.

### Biochemical parameters

Blood samples were collected from the cava vein after completing the hemodynamic study. Glucose, creatinine, bilirubin, aspartate aminotransferase, ALT, alkaline phosphatase (ALP), CK, total cholesterol, high-density lipoprotein (HDL), low density lipoprotein (LDL), triglycerides and albumin were measured using standard methods at the Hospital Vall d'Hebron CORE lab. Insulin was measured using an ELISA kit (EMD Millipore, Billerica, MA, USA). Insulin resistance was estimated by applying the homeostasis model of insulin resistance index (HOMA-IR):

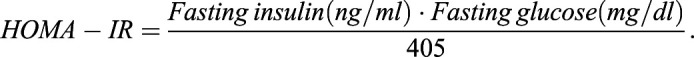


Hepatic and muscular toxicity due to statin treatment was defined based on ALT and CK levels in vehicle rats.

### Hemodynamic measurements

Ninety minutes after the last treatment administration, fasted rats were intraperitoneally anesthetized with ketamine hydrochloride (100 mg/kg) plus midazolam (5 mg/kg), and body temperature was maintained at 37°C for continuous recording of hemodynamic parameters. Mean arterial pressure (MAP, mmHg) was measured by catheterization (polyethylene PE-catheter, PE50) of the femoral artery, and PP (mmHg) was assessed by ileocolic vein catheterization using highly sensitive pressure transducers (Harvard Apparatus, Holliston, MA, USA). Superior mesenteric artery blood flow (SMABF, ml/[min×100 g]) and PBF (ml/[min×100 g]) were measured using a perivascular ultrasonic transit-time flow probe (1 mm diameter, Transonic Systems Inc, Ithaca, NY, USA). Superior mesenteric artery resistance (SMAR, mmHg/ml×min×100 g) and IHVR (mmHg/ml×min×100 g) were calculated as [(MAP-PP)/SMABF] and (PP/PBF), respectively.

### Histological analysis

#### Hematoxylin-eosin staining

Liver samples were fixed in 4% formaldehyde for 24 h, embedded in paraffin and sectioned in 4 μm thick slides. Hematoxylin-eosin was used to assess liver. Samples were evaluated by an expert liver pathologist blinded to animal interventions.

NAS was used to quantify NAFLD activity, obtained from the unweighted sum of the histological components: steatosis (0-3), lobular inflammation (0-3) and hepatocellular ballooning (0-2) (Kleiner et al., 2005). Table S2 shows CRN quantification system definitions. The diagnosis of histological NASH was made as per current standards, based on the concurrence of steatosis, ballooning and inflammation, and a NAS≥3.

#### Oil red O staining

Liver samples sections were frozen and sectioned into 8 μm slices, fixed in 4% formaldehyde and stained with Oil Red O for 10 min. Lipid droplets were quantified in five images (magnification 20×) of each section using Fiji software (Schindelin et al., 2012).

### LSEC and HSC isolation

LSECs and HSCs were isolated from rat livers as described previously (Marrone et al., 2013).

### LSEC isolation

LSECs were isolated from HFGFD-Veh (*n*=3) and HFGFD-Am^lo^ (*n*=3) rat livers. Livers were perfused with collagenase through the portal vein for 10 min at a flow rate of 20 ml/min at 37°C with Hanks' balanced salt solution (HBSS) without calcium and magnesium, containing 12 mM HEPES (pH 7.4), 0.6 mM EGTA and 1.6% bovine serum albumin. The livers were then perfused through the portal vein for 30 min at a flow rate of 5 ml/min at 37°C with 0.01% collagenase A, HBSS containing 12 mM HEPES (pH 7.4) and 4 mM CaCl_2_, and then excised and *in vitro* digested for 10 min at 37°C, also with the same buffer. Resulting cells were filtered through a 100 µm nylon filter, collected in cold Krebs buffer and centrifuged at 50 ***g*** for 5 min to eliminate hepatocytes. The supernatant was then centrifuged at 800 ***g*** for 10 min, and the pellet was resuspended in ice-cold PBS and centrifuged in a two-phase Percoll gradient (25%/50%). The central fraction containing LSECs and Kupffer cells (KCs) was collected, washed with PBS, resuspended in LSEC medium [RPMI with 10% fetal bovine serum (FBS), 2 mM L-glutamine, 1% penicillin/streptomycin, 1% amphotericin B, 0.1 mg/ml heparin and 0.05 mg/ml endothelial cell growth supplement (ECGS)] and seeded in a non-coated plate for 30 min at 37°C (5% CO_2_). KCs attached to the plate were discarded and the non-adherent LSECs were seeded in collagen-coated culture plates, incubated for 45 min (37°C, 5% CO_2_) and washed afterwards. The resulting cells were incubated at 37°C in a humid atmosphere with 5% CO_2_ in LSEC medium (RPMI with 10% FBS, 2 mM L-glutamine, 1% penicillin/streptomycin, 1% amphotericin B, 1% heparin and 1% ECGS).

### HSC isolation

HSCs were isolated from CD-Veh (*n*=4), HFGFD-Veh (*n*=4), HFGFD-At (*n*=5), HFGFD-Am^lo^ (*n*=4) and HFGFD-AtAm^lo^ (*n*=5) rat livers. Briefly, livers were perfused with pronase, collagenase and DNase through the portal vein for 10 min at a flow rate of 20 ml/min at 37°C with Gey's balanced salt solution (GBSS). The livers were then perfused through the portal vein for 30 min at a flow rate of 5 ml/min at 37°C, excised and *in vitro* digested for 10 min at 37°C, also with pronase, collagenase and Dnase. The resulting cells were filtered and centrifuged at 50 ***g*** for 5 min to eliminate the hepatocytes. The supernatant was then centrifuged at 800 ***g*** for 10 min and the pellet was resuspended in ice-cold GBSS and centrifuged in Optiprep gradient (11%). The fraction containing HSCs was collected, washed with GBSS, resuspended in HSC medium (Iscove's modified Dulbecco's medium with 10% FBS, 2 mM L-glutamine, 1% penicillin/streptomycin and 1% amphotericin B) and seeded in a non-coated plate at 37°C (5% CO_2_) overnight and then washed.

### Western blot analysis

Livers were perfused with saline for exsanguination and samples were directly frozen in liquid nitrogen, crushed to powder and homogenized in Triton X-100-lysis buffer [25 mM Tris/HCl (pH 7.6), 137 mM NaCl, 2.7 mM KCl, 20 mM NaF, 10 mM Na_4_P_2_O_7_, 10 nM okadaic acid, 2 mM Na3VO4, 2 µg/ml antipain, 2 µg/ml aprotinin, 2 µg/ml chymostatin, 2 µg/ml leupeptin, 2 µg/ml pepstatin A, 2 µg/ml trypsin inhibitor, 40 µg/ml phenylmethylsulfonylfluoride, and 10% (v/v) Triton X-100]. LSECs were washed with PBS and lysed using Triton X-100-lysis buffer. Homogenized livers and cell lysates were sonicated and centrifuged at 14,000 ***g*** at 4°C for 10 min. Supernatant protein concentration was assessed using a BCA Protein Assay Kit (Thermo Fisher Scientific, Rockford, IL, USA). Equal amounts of protein were run on a 10% SDS-PAGE. Proteins were blotted onto a polyvinylidene difluoride membrane (Thermo Fisher Scientific, Waltham, MA, USA). Membranes were blocked in 5% phosphoblocker (Cell Biolabs, San Diego, CA, USA) and incubated with the relevant primary antibody (Table S3) overnight at 4°C. Then, membranes were incubated with the corresponding secondary peroxidase-coupled antibody for 1 h at room temperature, developed using an ECL kit (GE Healthcare) and quantified by Image Studio Lite (Lincoln, NE, USA). Glyceraldehyde-3-phosphate dehydrogenase (GAPDH, 1/5000, Ambion, Austin, TX, USA) was used as loading control.

When detecting the phosphorylated and the total protein in the same membrane (for Akt and eNOS), the detection of the phosphorylated protein was performed first. Membranes were then stripped by incubating them with Restore WB Stripping Buffer (Thermo Fisher Scientific, Rockford, IL, USA) for 30 min at 55°C. Thereafter, they were blocked again and incubated with the primary and secondary antibody as described above to detect total protein levels. In this case, the ratio between the intensities of the phosphorylated and the total protein (P-Akt/Akt or p-eNOS/eNOS) were calculated without requiring GAPDH normalization.

### Primary LSEC culture with conditioned medium from HSCs

Endothelin-1-related paracrine effects between LSECs and HSCs were determined through a conditioned medium study (Dirscherl et al., 2020). HSCs were isolated from healthy rats and allowed to attach overnight in 12-well plates at confluency. Culture medium was replaced by fresh medium (serum free RPMI), and incubated for 10 min with the endothelin receptor A specific antagonist BQ-123 (10 µM) or vehicle. Then, ET-1 (10 nM) was added to the medium and incubated for 10 min more. After incubation, the conditioned medium was transferred to overnight serum-starved LSECs (isolated from healthy rats and seeded in 12-well plates at confluency) previously incubated with endothelin receptor B-specific antagonist BQ-788 (1 µM) or vehicle for 15 min, and incubated for 30 min.

### HSC collagen gel contraction assay

Twenty four-well plates were used to examine the contractile capacity of HSCs as described previously with slight modifications (Rockey and Weisiger, 1996). In brief, 1.5 mg/ml collagen (Collagen R Solution 0.2% SERVA, Heidelberg, Germany) gels were prepared using 1 N NaOH for pH adjustment. An aliquot of 500 µl of the solution was added to each well and incubated at 37°C for at least 1 h to allow gelatinization. Freshly isolated HSCs (2×10^6^) from the study groups were seeded on each gel in 1 ml of medium and incubated overnight. Cells were starved for 1 h, and the medium was then replaced by medium with 10% FBS, or medium with 10% FBS and 100 nM ET-1. The tip of a 200 μl pipette was used to gently detach the gel from the plates. After incubation for 24 h, the areas of the gels were measured using Fiji software (Schindelin et al., 2012). Triplicates of each condition were made, with cells isolated from at least three rats from each study group.

### RNA extraction and gene expression

RNA extraction was performed with HSCs isolated from the study groups using an RNeasy Mini Kit (Qiagen, Venlo, The Netherlands) following the manufacturer's instructions and reverse transcribed to cDNA (High Capacity cDNA Reverse Transcription Kit, Thermo Fisher Scientific). cDNA was added to Taqman universal PCR master mix plus the specific probe (Table S4) and loaded into 384-well plates (Thermo Fisher Scientific). qRT-PCR was performed using a 7900HT Fast Real-Time PCR system (Thermo Fisher Scientific). The relative gene expression was normalized to GAPDH. Data were analysed using the Relative Quantification qPCR Application in Thermo Fisher Cloud.

### Statistical analysis

Statistical analysis was performed using the IBM SPSS 20.0 statistical package (IBM, New York, USA). Data are reported as mean±s.e.m. and statistical significance was determined using Student's *t*-test, *U*-Mann–Whitney test or one-way ANOVA, followed by a parametric or non-parametric post-hoc test according to variance homogeneity determined by Levene's test. *P*<0.05 was considered statistically significant.

## Supplementary Material

Supplementary information
