# Supplementary material for: Synergic effect of atorvastatin and ambrisentan on sinusoidal and hemodynamic alterations in a rat model of NASH
Source: Dis Model Mech. 2021 May 20;14(5):dmm048884. doi: 10.1242/dmm.048884 (PMC8188885; doi:10.1242/dmm.048884)
Supplement: Supplementary information [file dmm-14-048884-s1.pdf]

Table S1. Serum biochemical parameters

	n	Glucose (mg/dL)	Creatinine (mg/dL)	Bilirubin (mg/dL)	AST (IU/L)	ALT (IU/L)	CK (IU/L)	AP (IU/L)	Cholesterol (mg/dL)	LDL (mg/dL)	HDL (mg/dL)	TGL (mg/dL)	Albumin (g/dL)
**CD-Veh**	7	128,83 ± 6,80	0,51 ± 0,06	0,11 ± 0,01	160,33 ± 21,21	34,71 ± 3,08	827 ± 103,94	120,43 ± 6,05	89,57 ± 8,11	29,57 ± 3,18	54,00 ± 5,17	28,5 ± 2,17	2,99 ± 0,07
**HFGFD-Veh**	6	151,67 ± 14,91	0,50 ± 0,03	0,12 ± 0,02	168,17 ± 20,08	73,00 ± 16,63***	751,17 ± 85,97	131,83 ± 8,56	79,83 ± 5,72	27,33 ± 2,93	47,67 ± 3,56	24,17 ± 2,7	2,95 ± 0,07
**HFGFD-At**	8	152,50 ± 16,26	0,66 ± 0,03*#	0,12 ± 0,02	128,88 ± 10,82	37,00 ± 2,73###	787,86 ± 97,28	130,00 ± 13,13	76,13 ± 3,45	25,25 ± 1,83	45,38 ± 2,78	27,25 ± 4,55	2,95 ± 0,08
**HFGFD-Am^{hi}**	7	152,57 ± 17,05	0,54 ± 0,04	0,11 ± 0,01	131,5 ± 9,48	42,71 ± 3,48##	882,83 ± 72,75	148,86 ± 10,02	79,43 ± 8,75	24,57 ± 2,64	47,71 ± 6,92	36,14 ± 3,92	3,06 ± 0,06
**HFGFD-AtAm^{hi}**	8	141,5 ± 10,65	0,57 ± 0,04	0,10 ± 0,02	127,88 ± 10,14	34,25 ± 2,47###	723,14 ± 105,17	139,00 ± 8,93	72,75 ± 5,85*	23,88 ± 2	42,13 ± 4,21	33,75 ± 4,6	2,90 ± 0,06
**HFGFD-Am^{lo}**	7	146,86 ± 17,95	0,59 ± 0,06	0,12 ± 0,01	171,29 ± 19,54	38,43 ± 3,67###	857,00 ± 123,09	127,86 ± 14,35	77,71 ± 3,56	24,14 ± 2,05	46,29 ± 2,83	36,57 ± 5,42	2,97 ± 0,03
**HFGFD-AtAm^{lo}**	8	133,13 ± 13,35	0,50 ± 0,03	0,14 ± 0,01	135,13 ± 18,10	39,75 ± 4,75###	797,63 ± 149,32	138,00 ± 12,97	73,75 ± 3,07*	23,50 ± 1,97	45,38 ± 2,06	23,75 ± 4,04	2,96 ± 0,05

Values are expressed as mean ± SEM. n, number of rats; AST, aspartate aminotransferase; ALT, alanine aminotransferase; CK, creatine kinase AP, alkaline phosphatase; TGL, triglycerides. CD (control diet); HFGFD-Veh (vehicle); HFGFD-At (10 mg/kg/day atorvastatin); HFGFD-Am^{hi} (30 mg/kg/day ambrisentan); HFGFD-AtAm^{hi} (10 mg/kg/day atorvastatin and 30mg/kg/day ambrisentan); HFGFD-Am^{lo} (2 mg/kg/day ambrisentan); HFGFD-AtAm^{lo} (10mg/kg/day atorvastatin and 2mg/kg/day ambrisentan). *$p < 0.05$, vs. CD; #$p < 0.05$ vs. HFGFD-Veh (ANOVA).

Table S2. ***NASH Clinical Research Network Scoring System Definitions (Kleiner et al., 2005)***

Item	Definition	Score
Steatosis	Low-to-medium power evaluation of parenchymal involvement by steatosis	
	<5%	0
	5-33%	1
	33-66%	2
	>66%	3
Lobular inflammation	Overall assessment of all inflammatory foci	
	No foci	0
	<2 foci per 200 x field	1
	2-4 foci per 200 x field	2
	>4 foci per 200 x field	3
Hepatocellular ballooning	None	0
	Few balloon cells	1
	Many cells/prominent ballooning	2

Disease Models & Mechanisms • Supplementary information

Table S3. Primary antibodies for Western blot.

Antibody	Company	Reference	Dilution
Rabbit polyclonal anti-P-eNOS (Ser1177)	Cell Signaling	#9571	1:250
Mouse monoclonal anti-eNOS	BD transduction Labs.	610296	1:500
Rabbit polyclonal anti-P-Akt (Ser473)	Cell Signaling	#9271S	1:500
Rabbit polyclonal anti-Akt	Cell Signaling	#9272S	1:500
Goat polyclonal anti-KLF2	Santa Cruz biotechnology	Sc-18690	1:200
Mouse monoclonal anti-ET-1	Abcam	Ab2786	1:250
Mouse monoclonal anti-GAPDH	Thermo Fisher Scientific	Am4300	1:5000

Phosphorylated endothelial nitric oxide synthase (P-eNOS), phosphorylated protein kinase B (P-Akt,), Kruppel-like factor 2 (KLF2), endothelin-1 (ET-1), and glyceraldehyde-3-phosphate dehydrogenase (GAPDH).

Disease Models & Mechanisms • Supplementary information

Table. S4. Primers used in qPCR.

Gene symbol	Gene name	Gene alias	Assay ID
Acta2	Actin, alpha 2, smooth muscle, aorta	αSMA	Rn01759928
Col1a1	Collagen, type I, alpha 1	COL1A1	Rn01463848
Des	Desmin		Rn00574732
Edn1	Endothelin 1	Et1	Rn00561129
Ednra	Endothelin receptor type A	ETRA, ET_A	Rn00561137
Ednrb	Endothelin receptor type B	ETRB, ET_B	Rn00569139
Mmp2	Matrix metallopeptidase 2		Rn01538170
Pdgfrb	Platelet derived growth factor receptor, beta polypeptide	PDGFRβ	Rn00709573

All probes are from Thermo Fisher Scientific.

Disease Models & Mechanisms • Supplementary information

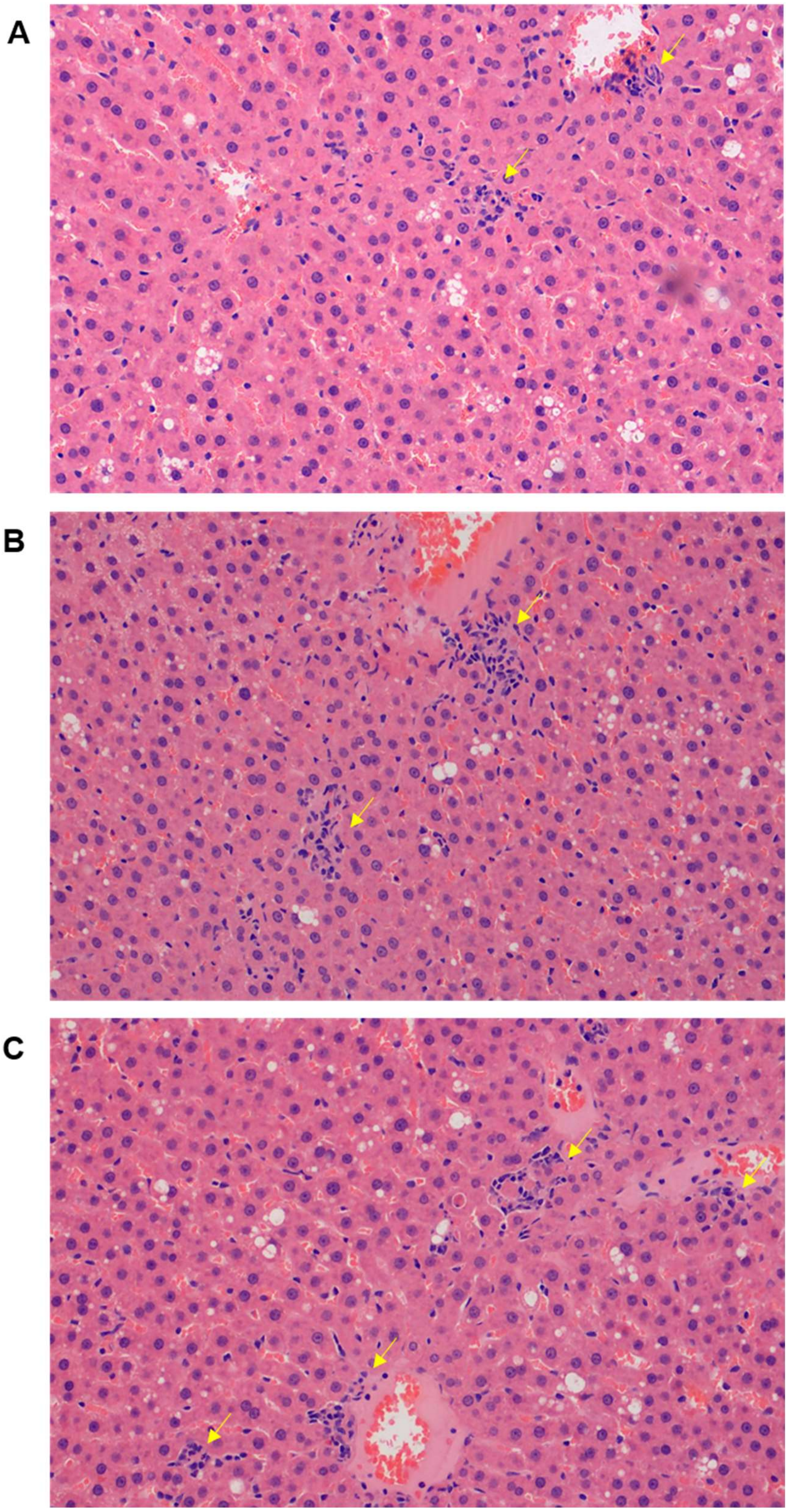

Figure S1. Representative images of lobular inflammation in HFGFD fed individuals. Images correspond to liver sections (20x magnification) stained with hematoxylin-eosin used to perform the histological evaluation. Yellow arrows point inflammatory foci.

Disease Models & Mechanisms • Supplementary information

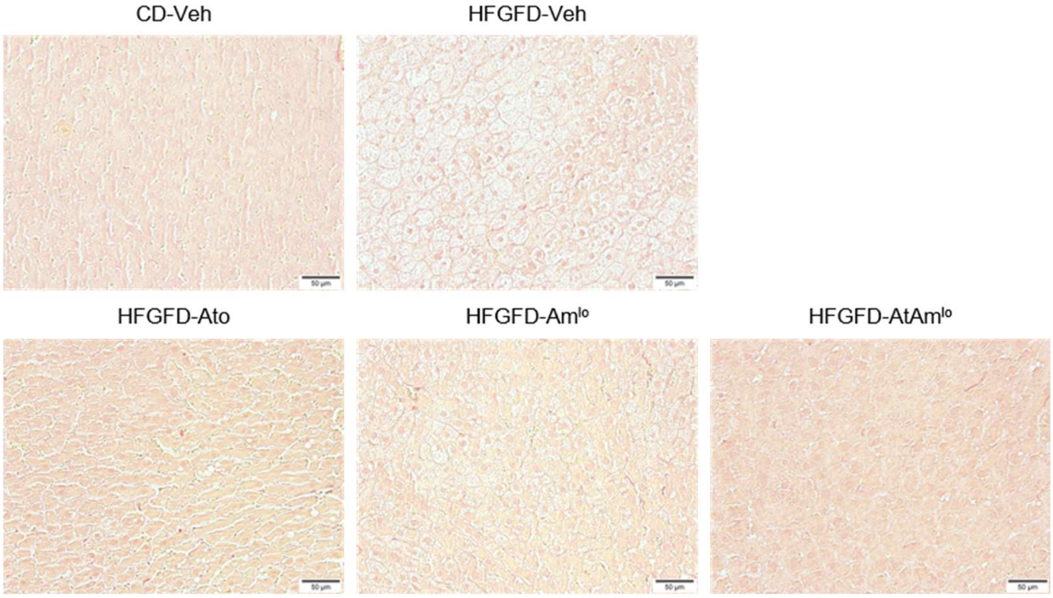

Figure S2. Representative images (20x magnification) of liver sections stained with Sirius Red to asses fibrosis.

Disease Models & Mechanisms • Supplementary information

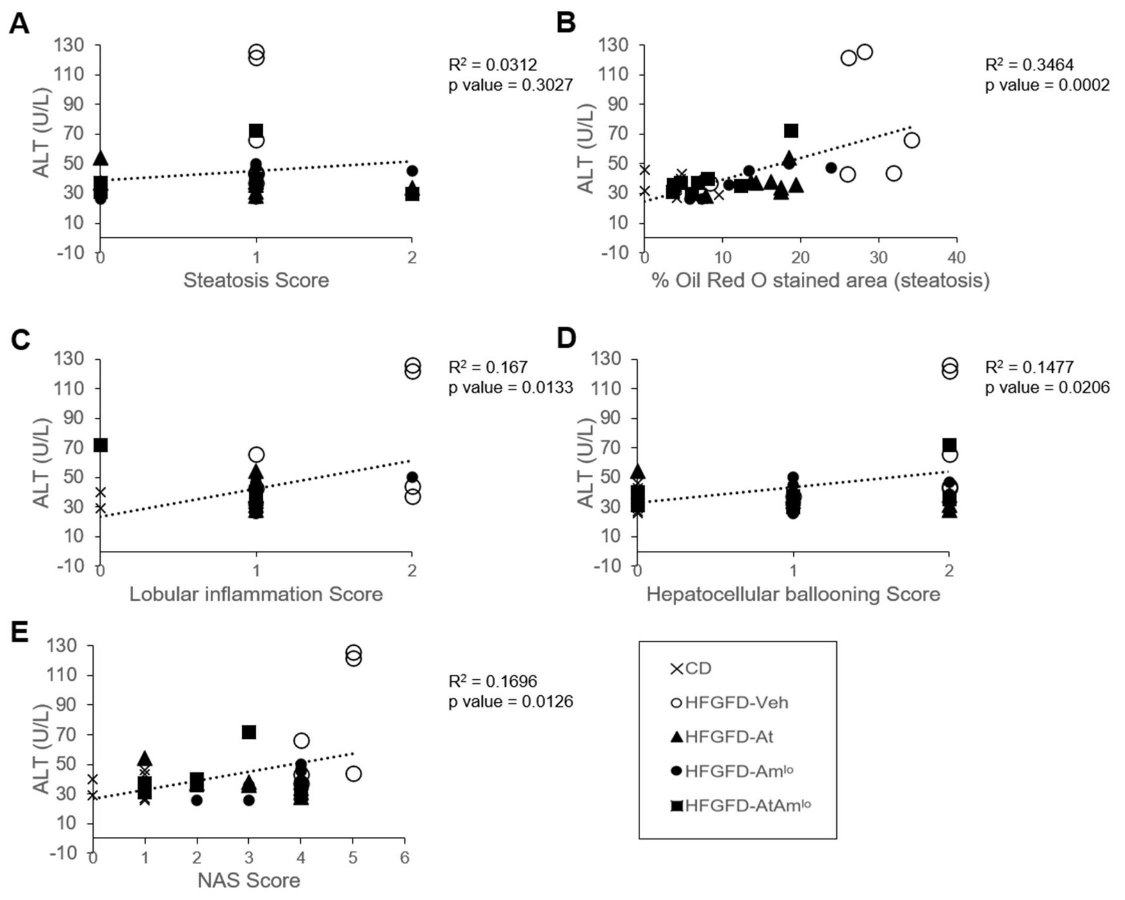

Figure S3. Correlations between serum ALT levels and (A) steatosis score (B) Oil Red O stained % area (C) lobular inflammation score (D) hepatocellular ballooning score and (E) NAFLD activity score (NAS).

Disease Models & Mechanisms • Supplementary information

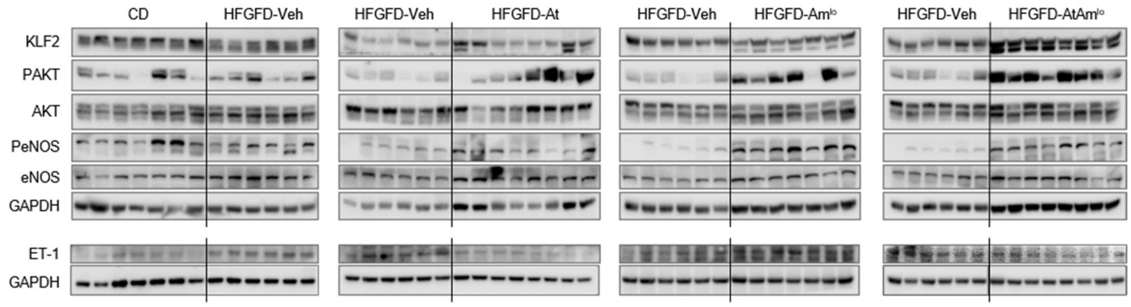

Figure S4. Complete Western blot images of P-Akt, phosphorylated protein kinase B, P-eNOS, phosphorylated endothelial nitric oxide synthase, KLF2, Kruppel-like factor 2; ET-1, endothelin-1; and GAPDH, glyceraldehydes 3-phosphate dehydrogenase from liver samples.
